# The Impact of Radiation Dose to Heart Substructures on Major Coronary Events and Patient Survival after Chemoradiation Therapy for Esophageal Cancer

**DOI:** 10.3390/cancers14051304

**Published:** 2022-03-03

**Authors:** Xin Wang, Nicolas L. Palaskas, Brian P. Hobbs, Jun-ichi Abe, Kevin T. Nead, Syed Wamique Yusuf, Joerg Hermann, Anita Deswal, Steven H. Lin

**Affiliations:** 1 Department of Radiation Oncology, The University of Texas MD Anderson Cancer Center, Houston, TX 77030, USA; samwang.md@gmail.com (X.W.); ktnead@mdanderson.org (K.T.N.); 2 Department of Radiation Oncology, Tianjin Medical University Cancer Institute and Hospital, National Clinical Research Center for Cancer, Tianjin 300060, China; 3 Department of Cardiology, The University of Texas MD Anderson Cancer Center, Houston, TX 77030, USA; nlpalaskas@mdanderson.org (N.L.P.); jabe@mdanderson.org (J.-i.A.); syusuf@mdanderson.org (S.W.Y.); adeswal@mdanderson.org (A.D.); 4 Department of Population Health, Dell Medical School, The University of Texas Austin, Austin, TX 78712, USA; brian.hobbs@austin.utexas.edu; 5 Department of Epidemiology, The University of Texas MD Anderson Cancer Center, Houston, TX 77030, USA; 6 Department of Cardiovascular Medicine, Mayo Clinic, Rochester, MN 55905, USA; herrmann.joerg@mayo.edu

**Keywords:** esophageal cancer, radiotherapy, heart substructure, major coronary events, survival

## Abstract

**Simple Summary:**

Whether it is necessary to evaluate the radiation exposure of cardiac substructures when making radiotherapy plans is one of the current research hotspots. In this cohort study of 355 patients with esophageal cancer, the radiation dose to key coronary substructures such as the left anterior descending artery V30_Gy_ and mean left main coronary artery was closely associated with major coronary events and overall patient survival, and showed better predictive value than the mean heart dose or heart V30_Gy_ recommended by current guidelines. Our findings suggest that, in addition to the whole heart, key coronary substructures should be contoured as organs at risk during radiotherapy plan optimization.

**Abstract:**

Background: There is a paucity of data regarding the association between radiation exposure of heart substructures and the incidence of major coronary events (MCEs) in patients with esophageal cancer (ESOC) undergoing chemoradiation therapy. We studied radiation dosimetric determinants of MCE risk and measured their impact on patient prognosis using a cohort of ESOC patients treated at a single institution. Methods: Between March 2005 and October 2015, 355 ESOC patients treated with concurrent chemoradiotherapy were identified from a prospectively maintained and institutional-regulatory-board-approved clinical database. Dose-distribution parameters of the whole heart, the atria, the ventricles, the left main coronary artery, and three main coronary arteries were extracted for analysis. Results: Within a median follow-up time of 67 months, 14 patients experienced MCEs at a median of 16 months. The incidence of MCEs was significantly associated with the left anterior descending coronary artery (LAD) receiving ≥30 Gy (V30_Gy_) (*p* = 0.048). Patients receiving LAD V30_Gy_ ≥ 10% of volume experienced a higher incidence of MCEs versus the LAD V30_Gy_ < 10% group (*p* = 0.044). The relative rate of death increased with the left main coronary artery (LMA) mean dose (Gy) (*p* = 0.002). Furthermore, a mutual promotion effect of hyperlipidemia and RT on MCEs was observed. Conclusion: Radiation dose to coronary substructures is associated with MCEs and overall survival in patients with ESOC. In this study, the doses to these substructures appeared to be better predictors of toxicity outcomes than mean heart dose (MHD) or whole-heart V30_Gy_. These findings have implications for reducing coronary events through radiation therapy planning.

## 1. Introduction

Radiation-induced heart disease following thoracic radiotherapy (RT) has long been reported in long-term survivors of breast cancer (BC) [1,2,3,4] and Hodgkin’s lymphoma (HL) [5,6,7]. Recently, it was found to be relatively common, with early onset, in patients with non-small cell lung cancer (NSCLC) [8,9,10] and esophageal cancer (ESOC), despite the high competing risk of death [11]. Radiation can induce a variety of pathological changes, including endothelial dysfunction, inflammation, thrombosis, and cardiac fibrosis, resulting in a variety of cardiotoxicities [12]. Coronary events are one of the important causes of cardiac mortality and morbidity among patients after RT [4,9,10,11,13].

Typically, the heart as a whole is considered as an organ at risk during thoracic RT, and the mean heart dose (MHD) has generally been used to assess the risk of coronary events in previous studies [4,14]. However, radiobiological responses in various heart substructures may be heterogeneous, and patients may have different cardiotoxicities depending on the radiation dose delivered to each individual cardiac substructure. In a group of patients with HL, Hahn et al. [15] found that the model engaging coronary artery variables was superior to the whole-heart model when analyzing ischemic cardiac events. Since the cardiac radiation exposure in ESOC patients is generally much higher than that in HL, an improved understanding of the dose–cardiotoxicity relationship while taking cardiac substructure volume exposure into consideration is particularly necessary to guide RT delivery.

Currently, the data are limited in terms of the association between radiation dose to heart substructures and major coronary events (MCEs). Our study aims to provide a detailed analysis within a modern cohort of ESOC patients treated with concurrent chemoradiotherapy at conventional radiation doses.

## 2. Materials and Methods

This cohort study comprised 355 patients with biopsy-confirmed esophageal adenocarcinoma or squamous cell carcinoma (SCC) that was treated with RT in prospective, single-institution biomarker or therapeutic trials in which detailed dosimetric data were maintained. Patients in this prospectively maintained database between March 2005 and October 2015 were analyzed (Supplementary Figure S1). All patients in our study underwent esophagogastroduodenoscopy (EGD) with endoscopic ultrasound, computed tomography (CT) of the chest and upper abdomen with contrast, brain imaging (CT or magnetic resonance imaging), and/or positron emission tomography (PET)/CT scans for staging, and were restaged according to the seventh edition of the American Joint Committee on Cancer TNM classification system [16]. Patients with distant metastatic disease, prior or concomitant malignancy, Eastern Cooperative Oncology Group performance status scale (ECOG) scores above 2, or those with incomplete clinical records were excluded. This study was approved by the Institutional Review Board of MD Anderson Cancer Center (protocol code: RCR02-542; date of initial approval: 13 September 2002; updated: 26 March 2021) and the Institutional Review Board waived the requirement for informed consent.

All patients were treated with concurrent chemoradiotherapy strategies, either as a pre-operative treatment or as a curative treatment. About one-third of patients (35.8%) received induction chemotherapy as part of a clinical trial or due to high-risk disease burden. Chemotherapy regimens typically consisted of fluoropyrimidine and platinum- or taxane-based compounds. Ivor Lewis esophagectomy was the most commonly employed surgical approach (79%). Radiotherapy was delivered with intensity-modulated radiation therapy (IMRT) or proton beam therapy (PBT), and the standard radiation dose was 50.4 Gy (relative biological effectiveness, RBE) in 28 fractions. When the patients were treated in free-breathing mode, four-dimensional computed tomography (CT) simulation was used to track tumor motion throughout the respiratory cycle. All normal structures were contoured on time-averaged CT scans. The IMRT plans were generated by the Pinnacle treatment planning system (version 9.0, Philips, Andover, MA); the PBT plans were generated by the Eclipse planning system (Varian Medical Systems, Liverpool, NY), 92% of which were completed using passive scattering proton therapy.

The whole heart, the atria, the ventricles, the left main (LMA), and the three main coronary arteries, namely the left anterior descending artery (LAD), the left circumflex artery (LCX), and the right coronary artery (RCA) were included in our analysis. An in-house multi-atlas contouring service (MACS) software program, the auto-contouring accuracy of which was previously verified [17], was used to automatically delineate the cardiac structures on the CT images for treatment planning. The accuracy and consistency of the heart substructures were reviewed for each patient by an experienced radiation oncologist, and necessary modifications were made according to the detailed guideline published by Feng et al. [18]. Dose-volume histograms of the heart were obtained from the delivered RT plan. Based on RTOG 0617 [19], we extracted the mean dose, volume receiving ≥ 5 Gy (V5_Gy_), volume receiving ≥ 30 Gy (V30_Gy_), and volume receiving ≥ 50 Gy (V50_Gy_) for the whole heart and for each cardiac substructure of interest for dosimetric analyses. Planning target volume (PTV) was a geometrical concept used for treatment planning, and defined as enlarged clinical target volume (CTV), which was created from the gross tumor volume (GTV) through volume expansion using individual margins.

The primary endpoint of this study was the occurrence of MCEs after RT [4], which was defined as a diagnosis of myocardial infarction (International Classification of Diseases, 10th Revision, codes 121 to 124), coronary revascularization, or death resulting from ischemic heart disease (codes 120 to 125). These events were verified by physicians who did not know the radiation dose distribution of the whole heart and its substructures and were independently reviewed by cardiologists based on the available source documentation. The time-to-failure endpoint was calculated as the duration from the RT start date to the first occurrence of MCE. Patients without MCEs were right-censored at the last point of contact. The secondary endpoint was overall survival (OS), which was defined as the time from diagnosis to death.

Descriptive statistics were used to characterize the baseline clinic-pathological characteristics of the patients. Overall survival was estimated using the Kaplan–Meier method with Greenwood’s formula for interval estimation. Cox proportional hazards regression was used to test for associations between patient clinicopathological characteristics and the study’s endpoints (MCE and OS). The competing risk regression method of Fine and Gray [20] was used to adjust the cumulative incidence of MCE for the competing risk of non-cardiac death. Statistical significance was conferred with a *p*-value < 0.05. Multiple regression analyses included factors identified with a *p*-value < 0.1 from univariate analysis. Maximally selected rank statistics further explored the utility of applying thresholds to variables identified as statistically significant in regression analysis to classify patients into low-risk versus high-risk groups. Thresholds were selected for individual variables to maximize the log-rank statistic. All analyses were conducted by R (version 3.6.2) and SPSS (version 25.0.0).

## 3. Results

### 3.1. Patient Characteristics and MCEs

**Figure 1:**
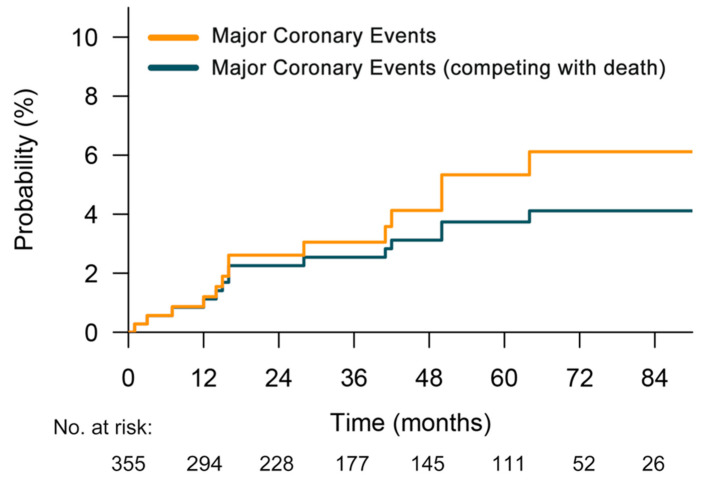
Cumulative incidence plot of major coronary events after chemoradiation (yellow) and major coronary events adjusted for the competing risk of death (green).

In total, 355 ESOC patients fulfilling the predefined criteria were included, with a median age of 62 years (interquartile range, 54 to 68 years). Among them, 89.6% were male and 88.2% were white. Baseline characteristics are summarized in Table 1. Within a median follow-up time of 67 months, 14 patients experienced MCEs at a median of 16 months (interquartile range, 12 to 42), which included myocardial infarction (*n* = 8), coronary artery bypass graft (*n* = 4), and atherosclerosis requiring coronary stent placement (*n* = 2). Details about these patients are provided in Supplementary Table S1. After accounting for non-cardiac death as a competing risk, the cumulative incidence of MCEs is shown in Figure 1. Additionally, detailed radiation dose distributions for the cardiac substructures are shown in Supplementary Figure S2.

### 3.2. Risk Factors for MCEs

On dosimetric analysis, LAD V30_Gy_ (%) (hazard ratio (HR) = 1.025; 95%CI, 1.001–1.050; *p* = 0.048) was significantly associated with MCEs (Supplementary Table S2). We identified the optimal curve cutoff value of LAD V30_Gy_ for predicting MCE at 10% (C-index, 0.57).

**Figure 2:**
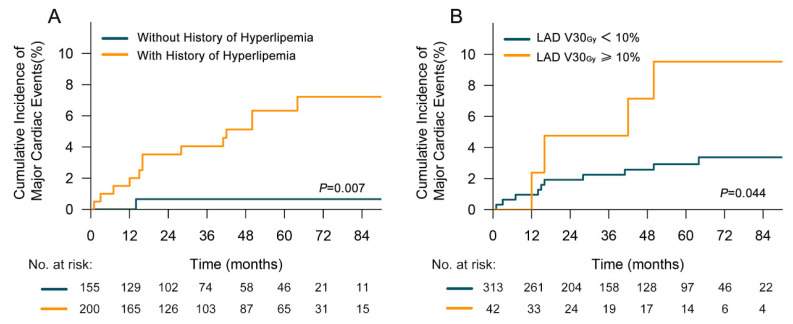
Cumulative incidence of major coronary events (with non-cardiac death as a competing risk) (**A**) for patients with or without a history of hyperlipidemia (**B**) for patients delivered to left anterior descending coronary artery (LAD) V30_Gy_ < 10% and ≥10%.

All clinicopathological factors were included in univariate analysis, and those associated with a *p* value < 0.1 were included in multivariate analysis. Significant correlations between MCE incidence and history of hyperlipidemia (Yes vs. No, HR = 10.522, 95%CI, 1.373–80.621; *p* = 0.023) as well as LAD V30_Gy_ (≥10% vs. <10%, HR = 3.589, 95%CI, 1.124–11.462; *p* = 0.031) were identified (Table 2). In patients undergoing RT, a history of hyperlipidemia was associated with an increased risk of developing an MCE (2-y rates, 3.5% vs. 0.7%; 5-y rates, 6.3% vs. 0.7%; *p* = 0.007) (Figure 2A). Compared with patients receiving LAD V30_Gy_ < 10%, the cumulative incidence of MCEs in patients with LAD V30_Gy_ ≥ 10% increased significantly (2-y rates, 4.8% vs. 2.0%; 5-y rates, 9.5% vs. 2.9%; *p* = 0.044) (Figure 2B).

### 3.3. Overall Survival

The median OS was 48 months for the entire group. As indicated by dosimetric analysis, the relative rate of death significantly increased with the mean LMA dose (HR = 1.014; 95%CI, 1.005–1.023; *p* = 0.002) and exhibited better predictive efficacy versus heart V30_Gy_ and MHD (Supplementary Table S3). The optimal cutoff value of the mean LMA dose was determined to be 20 Gy (C-index, 0.56).

**Figure 3:**
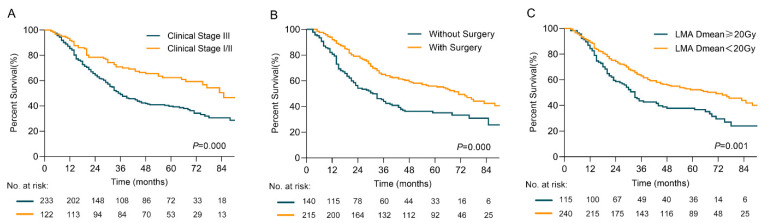
Overall survival rates (**A**) for patients with clinical stage I/II or III (**B**) for patients with or without surgery (**C**) for patients with 20 Gy mean left main coronary artery (LMA) dose cutoff.

Clinical stage (III vs. I/II; HR = 1.850; 95%CI, 1.332–2.571; *p* < 0.001), surgery (Yes vs. No; HR = 0.542; 95%CI, 0.407–0.723; *p* < 0.001), and mean LMA dose (≥20 Gy vs. <20 Gy; HR = 1.488; 95%CI, 1.108–1.998; *p* = 0.008) were identified as independent prognostic factors for OS (Table 3). Patients with an early clinical stage (I/II vs. III; 2-y rates, 78.5% vs.64.3%; 5-y rates, 62.4% vs. 39.7%; *p* < 0.001), surgical treatment (Yes vs. No; 2-y rates, 79.1% vs. 54.2%; 5-y rates, 55.9% vs. 35.1%; *p* < 0.001), and a mean LMA dose < 20 Gy (mean LMA dose < 20 Gy vs. ≥20 Gy; 2-y rates, 74.3% vs. 58.6%; 5-y rates, 52.3% vs. 37.8%; *p* = 0.001) demonstrated prolonged OS (Figure 3A–C).

## 4. Discussion

To our knowledge, this is the first study to demonstrate the relationship between MCEs and the radiation exposure of the cardiac substructures in a large cohort of ESOC patients. Our previous study in ESOC, in which the heart was analyzed as an entire organ, showed the correlation between cardiac toxicity and RT. [11]. In this study, we showed that the radiation doses to coronary substructures such as the LAD and LMA are important predictors of subsequent MCEs and OS, and are superior in this prediction compared with the dose to the whole heart as the method of prediction. Our results are consistent with a recent report in a large cohort of non-small cell lung cancer (NSCLC) patients treated with chemoradiation [21]. In that study, the authors found that the LAD V15_Gy_ ≥10% was strongly predictive of major adverse cardiac events (MACE), including coronary and heart failure events, cardiac death (adjusted HR 13.9; 95% CI, 1.23–157.21), and all-cause mortality. The dose cutoff for ESOC being higher than that for NSCLC (V30_Gy_ for ESOC vs. V15_Gy_ for NSCLC) may be related to the underlying comorbidities of patients with NSCLC vs. those with ESOC. These studies stress the importance of evaluating and avoiding radiation dosing to the key coronary substructures during RT planning for thoracic cancers such as ESOC and NSCLC.

At present, the NCCN Clinical Practice Guidelines in Oncology for Esophageal and Esophagogastric Junction recommended MHD and heart V30_Gy_ to be the primary indices for the risk assessment of cardiac toxicity. However, highly inhomogeneous doses to the small volumes of the heart may result in diverse heart injuries. Nilsson et al. [22] demonstrated through coronary angiography that the location and severity of coronary artery stenoses were associated with the anticipated hotspot areas for radiation in women with breast cancer. Van et al. [14] found that the MHD-based normal tissue complication probability (NTCP) model for acute coronary events could be improved in terms of calibration and discrimination by replacing MHD with LV-V5_Gy,_. A recent publication from Canada evaluating ischemic-only late cardiotoxicity in a group of HL patients indicated that the best predictive model included age, LAD V5_Gy_, and LCX V20_Gy_ as variables [15]. In our study, LAD V30_Gy_ exhibited better prediction of MCEs than either MHD or heart V30_Gy_, and patients with LAD V30_Gy_ ≥10% had a significantly increased risk of MCEs, which occurred notably earlier, at a median of 16 months after RT. Accordingly, we recommend that key coronary substructures such as the LAD should be contoured as organs at risk, along with the whole heart, for RT plan optimization.

The other major finding of interest in our study was that among several cardiac risk factors and pre-existing coronary disease, hyperlipidemia was the only independent predictor of MCEs. Hyperlipidemia is a known risk factor for atherosclerosis and increases the incidence of coronary events [23]. Radiation exposure tends to accelerate this process. Mancuso et al. [24] showed that both chronic low dose rate and acute exposure of the coronary arteries to irradiation accelerate atherosclerosis in apolipoprotein E-deficient (ApoE−/−) mice. In the same model of spontaneous atherogenesis, Hoving et al. [25] also observed such an expedited process in the carotid arteries. Notably, in contrast to ApoE−/− mice, none of the irradiated or control wild-type C57BL/6J mice developed atherosclerotic plaques within the 30-week follow-up period. These studies and others [26] that showed a mutual promotion effect between hyperlipidemia and RT on coronary events support the findings of our study. In addition to reducing radiation exposure to the critical coronary substructures, we suggest that patients with a history of hyperlipidemia may benefit significantly from optimized lipid management during and after RT.

Accumulating evidence shows that an excessive cardiac dose potentially contributes to cardiotoxicity, which is indicative of poor OS [9,10,11,27,28]. In the Radiation Therapy Oncology Group (RTOG) 0617 study [19], 74 Gy delivered in 2 Gy daily fractions for patients with stage III NSCLC was no better than 60 Gy, and even potentially harmful, with corresponding increases in heart V5_Gy_ and heart V30_Gy_ that were both significantly associated with a greater risk of death. A close relationship between radiation exposure of the heart and OS was also found in our study. We further demonstrated that the radiation dose to the key cardiac substructures, such as LMA, performed as a better predictor of OS than MHD or heart V30_Gy_. Sparing these key substructures, in particular when making RT plans, could potentially improve patient outcomes.

This study should be interpreted in the context of some limitations. First, we were limited by the nature of a retrospective study. Although the events were reviewed from the patients’ medical records and adjudicated by cardiologists, we may have undercounted the incidence of MCEs if some of them occurred elsewhere and were not documented in the electronic medical records at MD Anderson Cancer Center (MDACC), or if the patients did not return for follow-up at MDACC. Second, attributed to potential inter-observer variation [29] and the impact of the motion of the heart and its substructures [30], the reconstruction of small structures such as coronary arteries might be less reliable. In order to minimize such uncertainties, we used a validated in-house software program for delineation, and the delineation was checked by a single radiation oncologist with a common contouring guideline. Lastly, given the smaller number of events, further subgroup analysis was not possible. We look forward to validating our findings in larger, prospective, cooperative group clinical trials in the future.

## 5. Conclusions

In conclusion, the radiation dose to the key coronary substructures was closely associated with MCEs and overall patient survival. Therefore, this parameter may be used as a better predictor of coronary events than MHD or heart V30_Gy_ for ESOC patients. RT plan optimization and dedicated dose constraints for these crucial substructures of the heart may be the best option to reduce cardiac injury and prolong patient survival. Moreover, hyperlipidemia was an aggravating factor for MCEs in the presence of RT in our study, which suggests that optimization of lipid management may be particularly important during and after RT, especially in patients with thoracic cancers such as ESOC.

## Figures and Tables

**Table 1:** Patients characteristics at baseline.

Characteristic	Total		Non-MCEs		MCEs		*p*-Value
	N	%	N	%	N	%	
Sex							0.647
Male	318	89.6	306	89.7	12	85.7	
Female	37	10.4	35	10.3	2	14.3	
Age							0.780
<65	222	62.5	214	62.8	8	57.1	
≥65	133	37.5	127	37.2	6	42.9	
ECOG							0.586
0	148	41.7	141	41.3	7	50.0	
1–2	207	58.3	200	58.7	7	50.0	
History of Smoking							0.564
Yes	236	66.5	228	66.9	8	57.1	
No	119	33.5	113	33.1	6	42.9	
BMI, kg/m^2^							0.587
<30	206	58.0	199	58.4	7	50.0	
≥30	149	42.0	142	41.6	7	50.0	
History of CAD							0.708
Yes	57	16.1	54	15.8	3	21.4	
No	298	83.9	287	84.2	11	78.6	
History of Hyperlipidemia							0.005
Yes	200	56.3	187	54.8	13	92.9	
No	155	43.7	154	45.2	1	7.1	
History of Hypertension							0.090
Yes	218	61.4	206	60.4	12	85.7	
No	137	38.6	135	39.6	2	14.3	
History of Diabetes							0.775
Yes	89	25.1	86	25.2	3	21.4	
No	266	74.9	255	74.8	11	78.6	
Tumor Location							0.180
Upper/Middle	38	10.7	35	10.3	3	21.4	
Distal/GEJ	317	89.3	306	89.7	11	78.6	
Pathology							0.065
Adenocarcinoma	314	88.5	304	89.1	10	71.4	
SCC	41	11.5	37	10.9	4	28.6	
Clinical T Stage (7th)							0.377
T1–2	37	10.4	37	10.9	0	0.0	
T3–4	318	89.6	304	89.1	14	100.0	
Clinical N Stage (7th)							0.773
N0	113	31.8	108	31.7	5	35.7	
N+	242	68.2	233	68.3	9	64.3	
Clinical Stage (7th)							1.000
Stage I/II	122	34.4	117	34.3	5	35.7	
Stage III	233	65.6	224	65.7	9	64.3	
Induction Chemotherapy							0.592
Yes	127	35.8	123	36.1	4	28.6	
No	228	64.2	218	63.9	10	71.4	
Surgery							0.578
Yes	215	60.6	208	61.0	7	50.0	
No	140	39.4	133	39.0	7	50.0	
Radiotherapy Modality							0.397
IMRT	234	65.9	223	65.4	11	78.6	
PBT	121	34.1	118	34.6	3	21.4	
PTV							1.000
<600 cc	173	48.7	166	48.7	7	50.0	
≥600 cc	182	51.3	175	51.3	7	50.0	
Platin-based Chemotherapy							0.581
Yes	144	59.4	137	40.2	7	50.0	
No	211	40.6	204	59.8	7	50.0	

Abbreviations: ECOG, Eastern Cooperative Oncology Group; BMI, body mass index; CAD, coronary artery disease; GEJ, gastroesophageal junction; SCC, squamous cell carcinoma; IMRT, intensity-modulated radiation therapy; PBT, proton beam therapy; PTV, Planning Target Volume.

**Table 2:** Univariable and multivariable analysis for time to earliest major coronary events.

Variable	Univariable Cox Regression Analysis			Cox Multivariable Regression		
	HR	95% CI	*p*-Value	HR	95% CI	*p*-Value
Sex, Male vs. Female	0.778	0.174–3.476	0.742			
Age, ≥65 vs. <65	1.394	0.483–4.023	0.539			
ECOG, 1–2 vs. 0	0.754	0.264–2.151	0.597			
Smoking History, Yes vs. No	0.693	0.240–1.997	0.497			
BMI, kg/m^2^, ≥30 vs. <30	1.290	0.452–3.681	0.634			
History of CAD, Yes vs. No	1.735	0.483–6.232	0.398			
History of Hyperlipidemia, Yes vs. No	9.748	1.275–74.532	0.028	10.522	1.373–80.621	0.023
History of Hypertension, Yes vs. No	4.247	0.950–18.991	0.058			
History of Diabetes, Yes vs. No	0.853	0.238–3.060	0.808			
Tumor Location, Upper/Middle vs. Distal/GEJ	2.767	0.768–9.956	0.119			
Pathology, SCC vs. Adenocarcinoma	3.889	1.216–12.436	0.022			
Clinical Stage, III vs. I/II	1.190	0.397–3.564	0.756			
Induction Chemotherapy, Yes vs. No	0.705	0.221–2.247	0.554			
Surgery, Yes vs. No	0.477	0.166–1.368	0.168			
Radiotherapy Technology, IMRT vs. PBT	1.817	0.507–6.516	0.360			
PTV, ≥600 cc vs. <600 cc	1.051	0.368–2.998	0.926			
Platin-based Chemotherapy, Yes vs. No	1.349	0.473–3.849	0.576			
LAD, V30_Gy_ ≥ 10% vs. <10%	3.101	0.972–9.895	0.056	3.589	1.124–11.462	0.031

Abbreviations: CI, confidence interval; HR, hazard ratio; ECOG, Eastern Cooperative Oncology Group; BMI, body mass index; CAD, coronary artery disease; GEJ, gastroesophageal junction; SCC, squamous cell carcinoma; IMRT, intensity-modulated radiation therapy; PBT, proton beam therapy; PTV, Planning Target Volume; LAD, left anterior descending coronary artery.

**Table 3:** Univariable and multivariable analysis for overall survival.

Variable	Univariable Cox Regression Analysis			Cox Multivariable Regression		
	HR	95% CI	*p*-Value	HR	95% CI	*p*-Value
Sex, Male vs. Female	1.584	0.935–2.683	0.087			
Age, ≥65 vs. <65	1.061	0.793–1.420	0.688			
ECOG, 1–2 vs. 0	0.980	0.739–1.299	0.887			
Smoking History, Yes vs. No	1.168	0.864–1.578	0.314			
BMI, kg/m^2^, ≥30 vs. <30	0..925	0.697–1.229	0.593			
History of CAD, Yes vs. No	1.585	1.115–2.252	0.010			
History of Hyperlipidemia, Yes vs. No	0.872	0.659–1.155	0.340			
History of Hypertension, Yes vs. No	1.364	1.015–1.832	0.039			
History of Diabetes, Yes vs. No	1.296	0.950–1.766	0.102			
Tumor Location, Upper/Middle vs. Distal/GEJ	1.220	0.783–1.902	0.379			
Pathology, SCC vs. Adenocarcinoma	1.364	0.896–2.076	0.148			
Clinical Stage, III vs. I/II	1.863	1.353–2.564	0.000	1.850	1.332–2.571	<0.001
Induction Chemotherapy, Yes vs. No	0.879	0.655–1.180	0.390			
Surgery, Yes vs. No	0.557	0.420–0.738	0.000	0.542	0.407–0.723	<0.001
Radiotherapy Technology, IMRT vs. PBT	1.047	0.776–1.411	0.764			
PTV, ≥600 cc vs. <600 cc	1.319	0.995–1.748	0.054			
Platin-based Chemotherapy, Yes vs. No	0.955	0.719–1.269	0.751			
Mean LMA Dose, ≥20 Gy vs. <20 Gy	1.594	1.196	0.001	1.488	1.108–1.998	0.008

Abbreviations: CI, confidence interval; HR, hazard ratio; ECOG, Eastern Cooperative Oncology Group; BMI, body mass index; CAD, coronary artery disease; GEJ, gastroesophageal junction; SCC, squamous cell carcinoma; IMRT, intensity-modulated radiation therapy; PBT, proton beam therapy; PTV, Planning Target Volume; LMA, left main coronary artery.

## Data Availability

The data that support the findings of this study are available from the corresponding author (S.H.L.) upon reasonable request.

## Supplementary Materials

The following supporting information can be downloaded at: https://www.mdpi.com/article/10.3390/cancers14051304/s1, Figure S1: CONSORT diagram; Figure S2: Distribution of median dose indices (A) for heart chambers (B) for left main and three main coronary arteries; Table S1: Details of patients with major coronary events; Table S2: Dosimetric univariable and multivariable analysis for time to earliest major coronary event; Table S3: Dosimetric univariable and multivariable analysis for overall survival.
