# Supplementary material for: The Impact of Radiation Dose to Heart Substructures on Major Coronary Events and Patient Survival after Chemoradiation Therapy for Esophageal Cancer
Source: Cancers (Basel). 2022 Mar 3;14(5):1304. doi: 10.3390/cancers14051304 (PMC8909404; doi:10.3390/cancers14051304)
Supplement: Supplementary file 1 [file cancers-14-01304-s001.zip › cancers-1552530-supplementary.pdf]

cancers

MDPI

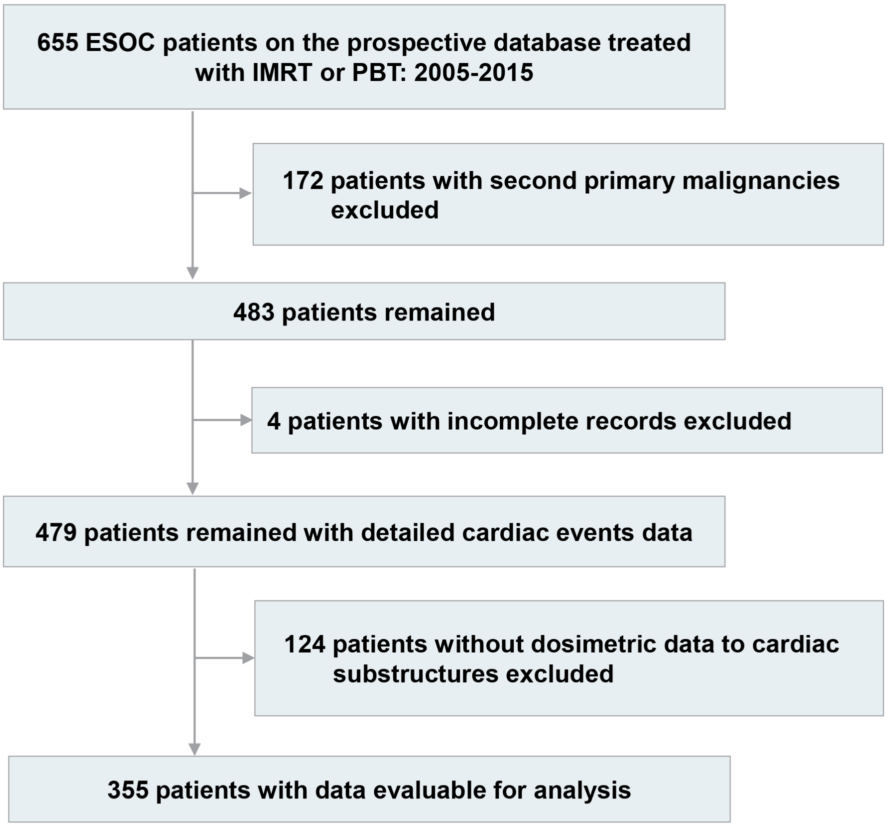

Figure S1. CONSORT diagram.

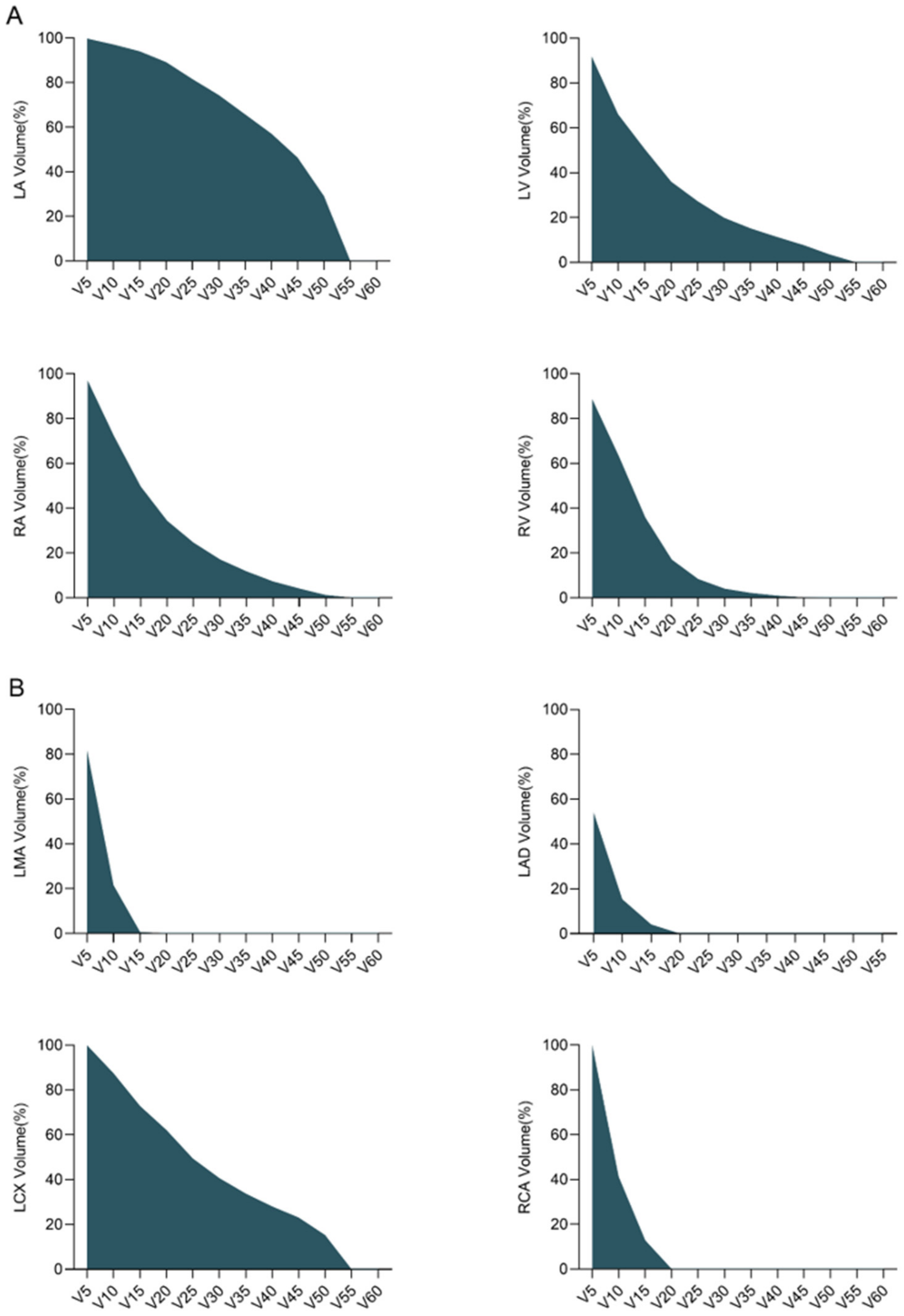

Figure S2. Distribution of median dose indices (**A**) for heart chambers (**B**) for left main and three main coronary arteries.

Table S1. Details of Patients with Major Coronary Events.

Age at Diagnosis	Sex	Time to Event (Month)	Event Details	Prior Cardiac Morbidity	Other Comorbidities
65	M	12	CABG	Myocardial infarction; Atrial fibrillation/Flutter; cardiomyopathy and cardiogenic shock	Hypertension; hyperlipidemia
56	M	42	Myocardial infarction	CAD	Hypertension; dyslipidemia; hyperlipidemia

73	M	3	Myocardial infarction	Atrial fibrillation	Hyperlipidemia
57	M	50	CABG	None	Hypertension; hyperlipidemia
68	M	16	Myocardial infarction	None	Hypertension; hyperlipidemia
59	M	7	Myocardial infarction	None	Diabetes; hypertension; hyperlipidemia
60	M	15	CABG	None	Diabetes; hypertension; hyperlipidemia
62	M	14	Stent in left anterior descending	None	Hypertension
64	M	50	Myocardial infarction	None	Hypertension; hyperlipidemia
65	F	16	CABG	None	Hypertension; hyperlipidemia
67	M	28	Coronary stent	None	Hypertension; hyperlipidemia
63	M	64	Myocardial infarction	None	Diabetes; hyperlipidemia; TIA
66	M	1	Myocardial infarction	CAD	Hypertension; hyperlipidemia; COPD; peripheral vascular disease
61	F	39	Myocardial infarction	None	Hypertension; hyperlipidemia

Abbreviations: CABG, Coronary Artery Bypass Grafting; CAD, Coronary Artery Disease; TIA, Transient Ischemic Attack; COPD, Chronic Obstructive Pulmonary Disease.

Table S2. Dosimetric Univariable and Multivariable Analysis for Time to Earliest Major Coronary Event.

	**Univariable Cox Regression Analysis**			**Cox Multivariable Regression**		
**Variable**	**HR**	**95%CI**	***p*-Value**	**HR**	**95%CI**	***p*-Value**
Heart $V5_{Gy}$ (%)	1.007	0.988-1.027	0.454			
Heart $V30_{Gy}$ (%)	1.021	0.994-1.048	0.132			
Heart $V50_{Gy}$ (%)	1.069	0.984-1.160	0.114			
Mean Heart Dose (Gy)	1.044	0.980-1.113	0.181			
LA $V5_{Gy}$ (%)	0.998	0.976-1.020	0.865			
LA $V30_{Gy}$ (%)	1.010	0.989-1.032	0.362			
LA $V50_{Gy}$ (%)	1.018	0.996-1.041	0.107			
Mean LA Dose (Gy)	1.021	0.973-1.072	0.404			
LV $V5_{Gy}$ (%)	1.006	0.987-1.025	0.552			
LV $V30_{Gy}$ (%)	1.006	0.978-1.034	0.694			
LV $V50_{Gy}$ (%)	1.040	0.979-1.104	0.201			
Mean LV Dose (Gy)	1.017	0.959-1.079	0.572			
RA $V5_{Gy}$ (%)	1.005	0.986-1.025	0.603			
RA $V30_{Gy}$ (%)	1.006	0.986-1.027	0.541			
RA $V50_{Gy}$ (%)	1.028	0.978-1.079	0.278			
Mean RA dose (Gy)	1.024	0.973-1.077	0.365			
RV $V5_{Gy}$ (%)	1.006	0.991-1.021	0.437			
RV $V30_{Gy}$ (%)	1.018	1.000-1.037	0.053			
RV $V50_{Gy}$ (%)	1.074	0.919-1.255	0.367			
Mean RV dose (Gy)	1.043	0.992-1.097	0.102			
LMA $V5_{Gy}$ (%)	1.005	0.993-1.016	0.436			
LMA $V30_{Gy}$ (%)	1.012	1.000-1.024	0.047			
LMA $V50_{Gy}$ (%)	0.989	0.890-1.099	0.838			
Mean LMA Dose (Gy)	1.032	0.999-1.065	0.058			
LAD $V5_{Gy}$ (%)	1.008	0.994-1.021	0.260			
LAD $V30_{Gy}$ (%)	1.025	1.000-1.050	0.048	1.025	1.001-1.050	0.048
LAD $V50_{Gy}$ (%)	0.566	0.004-87.235	0.825			
Mean LAD Dose (Gy)	1.054	0.996-1.117	0.072			
LCX $V5_{Gy}$ (%)	1.005	0.982-1.028	0.687			
LCX $V30_{Gy}$ (%)	1.008	0.989-1.026	0.413			

LCX $V50_{Gy}$ (%)	1.017	0.988-1.046	0.255
Mean LCX Dose (Gy)	1.020	0.973-1.069	0.418
RCA $V5_{Gy}$ (%)	1.005	0.992-1.018	0.488
RCA $V30_{Gy}$ (%)	1.006	0.985-1.028	0.584
RCA $V50_{Gy}$ (%)	0.610	0.004-104.450	0.851
Mean RCA Dose (Gy)	1.025	0.976-1.077	0.325

Abbreviations: CI, confidence interval; HR, hazard ratio; LA, Left Atrium; LV, Left Ventricle; RA, Right Atrium; RV, Right Ventricle; LMA, Left Main Artery; LAD, Left Anterior Descending Artery; LCX, Left Circumflex Artery; RCA, Right Coronary Artery.

Table S3. Dosimetric Univariable and Multivariable Analysis for Overall Survival.

	OS					
	**Univariable Cox Regression Analysis**			**Cox Multivariable Regression**		
**Variable**	**HR**	**95%CI**	***p*-Value**	**HR**	**95%CI**	***p*-Value**
Heart $V5_{Gy}$ (%)	1.002	0.997-1.007	0.386			
Heart $V30_{Gy}$ (%)	1.010	1.002-1.018	0.019			
Heart $V50_{Gy}$ (%)	1.023	0.998-1.049	0.075			
Mean Heart Dose (Gy)	1.018	1.000-1.035	0.046			
LA $V5_{Gy}$ (%)	1.003	0.996-1.009	0.427			
LA $V30_{Gy}$ (%)	1.006	1.000-1.011	0.039			
LA $V50_{Gy}$ (%)	1.006	0.999-1.012	0.078			
Mean LA Dose (Gy)	1.011	0.999-1.023	0.074			
LV $V5_{Gy}$ (%)	1.000	0.996-1.005	0.871			
LV $V30_{Gy}$ (%)	1.005	0.998-1.013	0.161			
LV $V50_{Gy}$ (%)	1.020	1.001-1.039	0.041			
Mean LV Dose (Gy)	1.007	0.991-1.023	0.369			
RA $V5_{Gy}$ (%)	1.001	0.996-1.006	0.604			
RA $V30_{Gy}$ (%)	1.003	0.997-1.009	0.289			
RA $V50_{Gy}$ (%)	0.994	0.972-1.017	0.613			
Mean RA dose (Gy)	1.009	0.995-1.023	0.224			
RV $V5_{Gy}$ (%)	1.001	0.998-1.005	0.441			
RV $V30_{Gy}$ (%)	1.007	1.000-1.013	0.041			
RV $V50_{Gy}$ (%)	1.039	0.991-1.091	0.114			
Mean RV dose (Gy)	1.014	1.000-1.029	0.045			
LMA $V5_{Gy}$ (%)	1.003	1.000-1.006	0.077			
LMA $V30_{Gy}$ (%)	1.005	1.001-1.008	0.011			
LMA $V50_{Gy}$ (%)	1.006	0.993-1.020	0.340			
Mean LMA Dose (Gy)	1.014	1.005-1.023	0.002	1.014	1.005-1.023	0.002
LAD $V5_{Gy}$ (%)	1.002	0.999-1.005	0.264			
LAD $V30_{Gy}$ (%)	1.011	1.001-1.021	0.028			
LAD $V50_{Gy}$ (%)	1.001	0.782-1.281	0.993			
Mean LAD Dose (Gy)	1.018	1.001-1.036	0.036			
LCX $V5_{Gy}$ (%)	1.002	0.996-1.008	0.444			
LCX $V30_{Gy}$ (%)	1.003	0.998-1.008	0.269			
LCX $V50_{Gy}$ (%)	1.004	0.995-1.012	0.400			
Mean LCX Dose (Gy)	1.008	0.995-1.021	0.225			
RCA $V5_{Gy}$ (%)	1.002	0.998-1.005	0.315			
RCA $V30_{Gy}$ (%)	1.004	0.997-1.010	0.264			
RCA $V50_{Gy}$ (%)	0.958	0.743-1.235	0.740			
Mean RCA Dose (Gy)	1.012	0.998-1.026	0.090			

Abbreviations: CI, confidence interval; HR, hazard ratio; LA, Left Atrium; LV, Left Ventricle; RA, Right Atrium; RV, Right Ventricle; LMA, Left Main Artery; LAD, Left Anterior Descending Artery; LCX, Left Circumflex Artery; RCA, Right Coronary Artery.
